# Clinical Determinants of HIV-1B Between-Host Evolution and their Association with Drug Resistance in Pediatric Patients

**DOI:** 10.1371/journal.pone.0167383

**Published:** 2016-12-01

**Authors:** Israel Pagán, Patricia Rojas, José Tomás Ramos, África Holguín

**Affiliations:** 1 Centro de Biotecnología y Genómica de Plantas (UPM-INIA) and E.T.S.I. Agrónomos, Universidad Politécnica de Madrid, Madrid, Spain; 2 HIV-1 Molecular Epidemiology Laboratory, Microbiology Department, Hospital Ramón y Cajal-IRYCIS and CIBER-ESP, Madrid, Spain; 3 Hospital Clínico San Carlos and Facultad de Medicina, Universidad Complutense de Madrid, Madrid, Spain; University of Pittsburgh, UNITED STATES

## Abstract

Understanding the factors that modulate the evolution of virus populations is essential to design efficient control strategies. Mathematical models predict that factors affecting viral within-host evolution may also determine that at the between-host level. Although HIV-1 within-host evolution has been associated with clinical factors used to monitor AIDS progression, such as patient age, CD4 cells count, viral load, and antiretroviral experience, little is known about the role of these clinical factors in determining between-host HIV-1 evolution. Moreover, whether the relative importance of such factors in HIV-1 evolution vary in adult and children patients, in which the course of infection is different, has seldom been analysed. To address these questions, HIV-1 subtype B (HIV-1B) *pol* sequences of 163 infected children and 450 adults of Madrid, Spain, were used to estimate genetic diversity, rates of synonymous and non-synonymous mutations, selection pressures and frequency of drug-resistance mutations (DRMs). The role and relative importance of patient age, %CD4, CD4/mm^3^, viral load, and antiretroviral experience in HIV-1B evolution was analysed. In the pediatric HIV-1B population, three clinical factors were primary predictors of virus evolution: Higher HIV-1B genetic diversity was observed with increasing children age, decreasing CD4/mm^3^ and upon antiretroviral experience. This was mostly due to higher rates of non-synonymous mutations, which were associated with higher frequency of DRMs. Using this data, we have also constructed a simple multivariate model explaining between 55% and 66% of the variance in HIV-1B evolutionary parameters in pediatric populations. On the other hand, the analysed clinical factors had little effect in adult-infecting HIV-1B evolution. These findings highlight the different evolutionary dynamics of HIV-1B in children and adults, and contribute to understand the factors shaping HIV-1B evolution and the appearance of drug-resistance mutation in pediatric patients.

## Introduction

HIV-1 populations are characterized by fast evolutionary rates and ample genetic diversity at both the within- and the between-host levels, which is primarily due to high virus replication rate, population size, and to the error-prone nature of its reverse transcriptase [1,2]. This high multi-level genetic variability has relevant implications for both HIV-1 evolution and AIDS development, as it is involved in the appearance of mutants that resist antiretroviral therapies (ART) and affects the rate of disease progression [3–6]. Hence, understanding the factors that modulate the evolution of HIV-1 populations at both the within- and the between-host levels may provide fundamental insights for developing more efficient strategies to control virus infection [reviewed by 4]. Despite the importance of this subject, such factors are still only partially understood.

Mathematical modelling of the determinants of RNA virus evolution, as HIV-1, has proposed that virus within- and between-host evolutionary dynamics are linked [7–9]. According to these models, factors that reduce within-host virus evolutionary rates, such as faster depletion of susceptible cells, lower age of infection at transmission, lower viral replication rates or control measures (e.g. vaccination, antiviral drugs), may increase between-host ones [7,8], therefore affecting virus population genetic diversity. Indeed, this association between within- and between-host evolution has been shown to explain the evolutionary dynamics of several host-virus interactions [8, 10–12]. For HIV-1, a number of clinical factors used to monitor disease progression have shown to determine virus within-host evolution in naïve patients: levels of CD4 count [13,14], viral load [15,16], virus exposure time [2,17], and age [3]. In treated patients, antiretroviral therapy (ART) has been also shown to promote HIV-1 adaptive evolution and fixation of drug resistance mutations (DRMs) during the course of the infection (e.g. [6,18]). These works have demonstrated that, individually, changes in various clinical factors are associated with HIV-1 evolution. However, this approach might represent an oversimplification, as during the course of an infection, or an epidemic, HIV-1 populations face changes in viral load, CD4 count, and ART experience that occur simultaneously. Therefore, a more complete understanding of the role of clinical factors in determining the evolution of HIV-1 populations would require of analyses that explore the relative importance of these clinical factors, and of their interactive effects. Such analyses are scant and do not generally considered evolution at the between-host level [3,4,19]. Thus, the role of clinical factors in HIV-1 between-host evolution remains largely unexplored [19].

Most of what is known about the clinical factors that shape HIV-1 evolution derives from analyses in adult patients. Comparatively little is known about the modulators of virus evolution in pediatric populations, even though over 3 million children currently live with HIV-1 [20]. HIV-1 genetic diversity in infected children may not necessarily evolve in the same way as in adults, as the course of virus infection is significantly different in these two groups of patients [21]. First, most HIV-1 infections in children occur perinatally, a time of relative immunologic immaturity [22]. Therefore, the immune system would exert a smaller selection pressure in HIV-1 pediatric than in adult populations, and fewer amino acid changes are expected to accumulate in children-infecting viral genomes at least in the early stages of infection [23]. Second, kinetics of viral load are also different: in children [24,25], but not in adults [16], plasma HIV-1 RNA remains elevated over the first two years of infection, and tends to decrease afterwards. Third, CD4 cells have been described to die at different rates in children and in adults [22]. Finally, toxicities, suboptimal therapies and/or lack of adherence result in a large proportion of HIV-infected children receiving multiple ARTs along their lives. Such low-efficiency treatments have an effect in the evolution of pediatric virus populations, as they result in the appearance of higher numbers of DRMs as compared with adults [18,26,27]. All these differences suggest that the relative importance of clinical factors in determining HIV-1 within-host evolution may vary between children and adult patients, which may affect virus between-host evolution. However, few studies have aimed at understanding the clinical determinants of the evolutionary rates and genetic diversity in pediatric HIV-1 populations [3,17,28]. Moreover, comparisons of the evolutionary dynamics of pediatric and adult HIV-1 populations are largely restricted to analyses in mothers and their children, generally involving small sample sizes [17,28].

To better understand the determinants of HIV-1 between-host evolution in pediatric populations, we analysed the role of several clinical factors associated with disease progression—CD4 count (both as % CD4 and as CD4/mm^3^), viral load, patient age/exposure time, and ART experience—in the between-host genetic diversity of the HIV-1 subtype B in the pediatric cohort of Madrid, Spain. The Madrid cohort of HIV-infected children and adolescents (from here on ‘pediatric cohort’) belongs to the Cohort of the Spanish Pediatric HIV Network (CoRISpe) [29]. This well described pediatric cohort has a number of characteristics that made it suitable to address the objectives of the present work. First, it includes patients with detailed follow up information of infection [30–32] and ART resistance [33–36]. This means that data on the above-mentioned clinical parameters, as well as virus sequences of the *pol* gene and frequency of drug resistance mutations, are available for a large number of individuals [34–36]. Second, the pediatric cohort includes patients of all ages, from new-borns to 21 years-old patients, many of which were infected perinatally [33]. This allowed analysing the effect of exposure time/age in the genetic diversity of HIV-1B populations. Third, the cohort includes naïve, as well as pre-treated individuals [32–35], so that the effect of ART experience in HIV-1B genetic diversity could be analysed. Finally, similarly detailed data on an HIV-infected adult cohort from Madrid is also available (from here ‘adult cohort’) [37,38], such that comparisons between the evolution of adult and pediatric HIV-1B populations from the same geographical area could be made. Using this unique data set, we analysed: i) if adult and pediatric HIV-1B populations differed in their level of between-host genetic diversity; ii) the relative importance of five clinical factor in determining the between-host evolution of the pediatric HIV-1B population; iii) how changes in these clinical factors, individually or in combination, were associated with the between-host genetic diversity of the pediatric HIV-1B population; and iv) whether changes in between-host genetic diversity were associated with the frequency of DRMs in the *pol* gene.

## Materials and Methods

### Study population

The Madrid cohort of HIV-infected children and adolescents was established in 2003. Since the beginning of the epidemic in Spain, 534 patients have been registered in the cohort, representing 51.4% of the infected pediatric population in the country. A total of 163 patients from this cohort were enrolled in the study, which were selected according to: 1) Availability of *pol* sequences collected between 1994 and 2015; 2) children being infected only by HIV-1B, to avoid bias in the estimation of evolutionary parameters due to heterotachy [39]; and 3) Geographical origin such that all patients were from Spain. Pediatric patients were infected through mother-to-child transmission. For comparison purposes, a dataset of previously published HIV-1B *pol* sequences from 450 infected adults from the Madrid cohort was used, including 60% of the mothers from infected children [37,38]. The study dataset included sequences derived from both ART-experienced and drug naïve patients. For the patients enrolled in the study, data on age, CD4 counts (percentage and cells/mm^3^), viral load (HIV-1B-RNA copies/ml of plasma, c/ml), and presence of drug resistance mutations at the moment of HIV-1B sampling for sequence determination were collected (S1 File).

### Ethics statement

All patients participating in the study gave their written informed consent. All children had written informed consent given from a parent or from the legal tutor (that is, from the legal guardian of the children) in the absence of the parents. This study was approved by the review board of the Hospital Universitario Ramón y Cajal Clinical Research Ethical Committee.

### Sequence data

Partial sequences of the *pol* gene from pediatric patients are described in [34], and sequences from adult patients were collected from different sources [33–35]. Sequences encompassed 334 amino acids: the 297 amino acids of the viral protease (*PR*), and a fragment coding for the first 37 amino acids of the reverse transcriptase (*RT*) (1,002 nucleotides, positions 2,255–3,256 of HXB2 subtype B reference strain, GenBank accession number K03455). A full list of GenBank accession numbers and year of isolation, the corresponding publication, and the relevant clinical parameters associated with each sequence is available in S1 File. Sequence alignments were constructed using MUSCLE 3.7 [40] and adjusted manually according to the amino acid sequences using Se-Al [41].

### Genetic diversity and selection pressures

Genetic diversity (*d*) was estimated as average pairwise nucleotide differences, using the Tamura-Nei Tamura-Nei, the Kimura 2-parameter and the composite likelihood nucleotide substitution models as implemented in MEGA 7 [42]. Since all models led to the same conclusions, for simplicity only values obtained using the Tamura-Nei model are presented. Standard errors (SE) of each measure were calculated using pairwise distances and were based on 1,000 bootstrap replicates for permutation tests. Selection pressures were measured as the ratio between the mean number of non-synonymous (*d*_N_) and synonymous (*d*_S_) nucleotide substitutions per site (*d*_N_/*d*_S_) using the single-likelihood ancestor counting (SLAC), the fixed effect likelihood (FEL), the random effects likelihood (REL), and the fast unbiased Bayesian approximation (FUBAR) methods implemented in the HyPhy package [43]. Because the four methods led to the same conclusions, only the FUBAR results are shown. In all cases, *d*_N_/*d*_S_ estimates were based on input neighbor-joining trees inferred using the MG94 nucleotide substitution model. Individual values of *d*_N_ and *d*_S_ were also obtained.

### Detection of recombination

Recombination breakpoints were detected using four different methods available in the RDP4 package: RDP, GENECONV, Bootscan, and Chimaera, and employing the default parameters [44]. Only recombination signals detected by all the methods (*P*<0.05) were considered as positive. The HIV-1B data sets used in this study did not contain recombinant sequences.

### Drug resistance mutations

Drug-resistance mutations among naïve patients were defined according to the mutation list for Transmitted Drug Resistances (TDR) surveillance as recommended by the WHO [45], and were mapped using the Calibrated Population Resistance tool [46]. Drug-Resistance Mutations (DRM) in treated patients were defined by the International AIDS Society-USA list (IAS) [47]. Drug susceptibility was estimated for protease inhibitors (PI), nucleoside and non-nucleoside reverse transcriptase inhibitors (NRTI and NNRTI, respectively), according to the HIVdb Interpretation Algorithm version 6.0.11 (Stanford University, Palo Alto, CA, USA) [48].

### Statistical analyses

Differences in genetic diversity, synonymous and non-synonymous diversity and selection pressures were calculated using parametric (General Linear Models, GLM) and non-parametric (Permutation tests) analyses. Since both approaches led to similar conclusions, for simplicity, only GLM analyses are shown. Variation in the frequency of sites under diversifying/purifying selection was analysed by Fisher’s exact test, with a Yates correction for small sample size when necessary [49]. The relative importance of each clinical factor on the pediatric cohort was estimated using a Principal Component Analysis. Children age (years), ART experience (presence or absence), % CD4 cells, CD4/mm^3^, and VL (c/ml) were scaled to zero mean and unit variance, inserted in a regression matrix and rotated to obtain the principal components (PCs). Significance thresholds for the load of each ecological factor on a PC were determined using a broken-stick model [50].

Multivariate tests were used to analyse the association between clinical factors and evolutionary parameters of the pediatric HIV-1B population [49]. A set of models that included a global model containing all clinical factors, and nested models that contained different combinations of the clinical factors was fitted for each evolutionary parameter using GLM (R library: lm), and linear mixed models (R library: nlme). Since both analyses yielded similar results, for simplicity only GLM data is presented. We used this approach because it allowed us to make inferences across a set of causal model structures for each evolutionary parameter [51]. Global and nested models containing clinical parameters as predictor variables were ranked according to Akaike’s Information Criteria (AIC) (R library: AICcmodavg), and the model with the lowest AIC score was selected as the best-ranked model. We calculated AIC Delta (Δ_*i*_), as the difference between the AIC of a given model and that of the best-ranked model. Delta quantifies how strongly models compete (Δ_*i*_ = 0 for best-ranked model; Δ_*i*_ = 1–2 indicates substantial empirical support; Δ_*i*_ = 4–7 indicates considerable less support; and Δ_*i*_ > 10 indicates no support [51]). For all evolutionary parameters, at least one model closely competed with the best-ranked model. Therefore, we also calculated a relative importance index (*ω*_*i*_) for each predictor using multi-model inference [51], following the expression: *ω*_*i*_ = exp(-0.5Δ_i_)/Σexp(-0.5Δ_i_). The larger the *ω*_*i*_, the greater the likelihood of the model given the data, relatively to the competing models. The relative importance of the predictor variables included in each model was calculated by hierarchical partitioning of the correlation coefficient (R library: relaimpo).

## Results

### Comparison of HIV-1B evolution and relative importance of clinical factors in pediatric and adult HIV-1B populations

Evolutionary parameters of the HIV-1B populations were estimated based on virus sequences from 163 pediatric, and the same number of adult, patients. Note that our sequence dataset included 450 adult patients (Table 1). In order to avoid sample size effects, minimize biases due to differences in CD4 count, viral load and ART experience; and maximise the ‘clock-likeness’ of the data, we created a ‘balanced’ dataset following [52]. Accordingly, we randomly removed sequences from the adult cohort to have the same number of instances than in the pediatric cohort, with the same naïve/treated proportion and with similar distribution of patients across CD4 count (both as CD4/mm^3^ and % CD4 cells) and viral load categories (Table 1). This randomization was repeated ten times and HIV-1B evolutionary parameters were estimated in each ‘balanced’ dataset. Average values of the ten ‘balanced’ datasets did not significantly differ between them and from the real data (S1 Table), indicating that they are representative of the HIV-1B population in adult patients. Data presented in the Results section correspond to average values for the ten ‘balanced’ datasets.

**Table 1:** Clinical and epidemiological parameters of the pediatric and adult HIV-infected populations used in this study.

Parameter	Children n = 163			Adults n = 450		
	Naive n = 30	Treated n = 133	Total n = 163	Naive n = 207	Treated n = 243	Total n = 450
**Gender [n]**						
Female	23	69	92	173	50	223
Male	7	64	71	18	19	37
Unknown	0	0	0	16	174	190
**Route of transmission [n]**						
Vertical	30	133	163	0	0	0
IDU	0	0	0	44	24	68
Sexual (MSM+Htsex)	0	0	0	122	24	146
Others	0	0	0	38	194	232
Unknown	0	0	0	3	1	4
**CD4+ T cell counts [%]**						
<25%	10	46	56	77	24	101
26–50%	10	64	74	105	153	258
>50%	1	1	2	0	9	9
Unknown	9	22	31	25	57	82
**CD4+ T cell counts [Cells/mm**^**3**^**]**						
<350	4	13	17	69	42	89
350–500	4	11	15	46	31	77
500–1000	7	51	59	47	88	157
1000–1500	2	15	17	11	10	21
>1500	4	14	18	9	15	24
Unknown	9	29	38	25	57	82
**Viral load [HIV-1 RNA-copies/ml]**						
<50	3	11	14	0	0	0
50–500	3	13	16	2	1	3
500–1,000	3	5	8	7	1	8
1,000–10,000	3	33	36	32	11	43
10,000–100,000	6	33	39	94	152	246
>100,000	11	19	30	47	21	68
Unknown	1	19	20	25	57	82
**DRM prevalence**^a^ **[n]**						
At least one DRM M	7	106	113	21	104	125
To PIs^b^	1	49	50	5	38	43
To NRTIs	4	92	96	14	92	106
To NNRTIs	5	63	68	8	66	74
None	22	27	49	186	139	325

IDU: injectable drug users; MSM: men who have sex with men; Htsex: heterosexual; DRM: drug resistance mutations; PI: protease inhibitors; NRTI: nucleoside reverse transcriptase inhibitors; NNRTI: non-nucleoside reverse transcriptase inhibitors.

^a^ DRM prevalence was determined following the WHO TRM list for ‘naïve’ patients [40], and the IAS–USA 2014 list for ‘treated’ patients [41].

^b^ Includes only major drug-resistance mutations at the viral protease.

**Fig 1:**
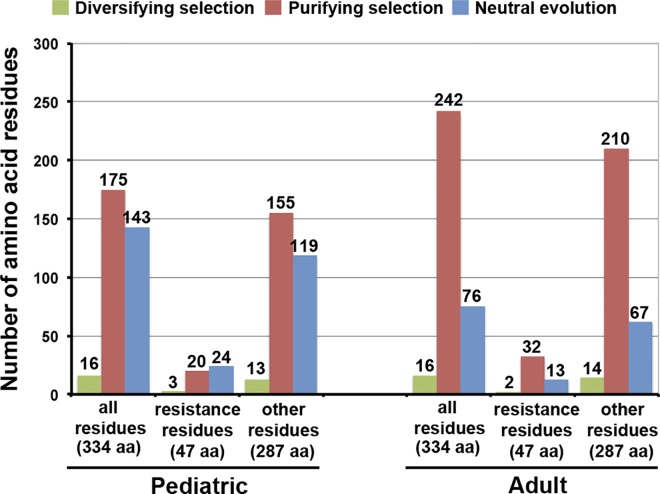
Selection pressures in amino acid residues of the *pol* protein. Number of amino acid residues (aa) of the protease (PR) and the retrotranscriptase (RT) proteins under purifying and diversifying selection, and under neutral evolution, in pediatric (A) and adult (B) HIV-1B populations. Data are presented either considering all sites or only those associated with drug resistance. Amino acid residues associated to antiretroviral resistance were chosen according to IAS-USA 2014 [47].

HIV-1B genetic diversity was lower in the pediatric than in the adult-infecting virus population (*d*: 0.054±0.002 *vs*. 0.058±0.002; *F* = 27.09, *P*<1x10^-5^), which was due to a lower synonymous diversity (*d*_S_: 0.145±0.001 *vs*. 0.165±0.002; *F* = 215.40, *P*<1x10^-5^) (Table 2). Non-synonymous diversity was similar in the pediatric and adult virus populations, and always smaller than synonymous diversity (*d*_N_: 0.031±0.004 *vs*. 0.030±0.002; *F* = 1.56, *P* = 0.834). Consequently, the two virus populations were under purifying selection, this being weaker in the pediatric than in the adult HIV-1B population (*d*_N_/*d*_S_: 0.212±0.009 *vs*. 0.180±0.002; *F* = 922.34, *P*<1x10^-5^) (Table 2). Accordingly, the majority of the amino acid sites were under purifying selection in both virus populations (Fig 1), but this proportion was higher in the adult than in the pediatric populations (242/334 *vs*. 175/334; *χ*^*2*^ = 28.65, *P*<1x10^-5^). The pediatric and adult HIV-1B populations had the same proportion of sites under diversifying selection (16/334). The lower number of sites under purifying selection in the pediatric virus population was accompanied by a higher frequency of sequences harbouring at least one DRM, as compared with the adult virus population (113/163 *vs*. 44/163; *χ*^*2*^ = 58.5, *P*<1x10^-5^). When only site known to be associated with drug resistance were considered, sites under purifying selection were more abundant in the adult- than in the pediatric-infecting virus population (32/47 *vs*. 20/47; *χ*^*2*^ = 6.20, *P* = 0.013). The proportion of drug-resistance sites under diversifying selection did not vary between pediatric and adult HIV-1B populations (3/47 vs. 2/47; *χ*^*2*^ = 0.05, *P =* 0.823) (Fig 1). Hence, HIV-1B evolution differs in pediatric and adult patients.

**Table 2:** Evolutionary parameters of the pediatric and adult HIV-1B populations based on a fragment of the *pol* gene (1,002 nt).

Parameters	Pediatric (age 0–21)			Adult (age >21)		
	Naïve	Treated	Total	Naïve	Treated	Total
**Evolutionary**						
*d*	0.047±0.002	0.061±0.002	0.054±0.002 §	0.059±0.001	0.056±0.001	0.058±0.002 *
*d*_N_	0.023±0.000	0.039±0.000	0.031±0.004 §	0.028±0.000	0.031±0.000	0.030±0.002
*d*_S_	0.143±0.002	0.147±0.000	0.145±0.001	0.178±0.003	0.152±0.002	0.165±0.002 §*
*d*_N_/*d*_S_	0.163±0.003	0.262±0.001	0.212±0.009 §	0.158±0.002	0.203±0.002	0.180±0.002 §*
**Clinical**						
% CD4	23.82±3.02	28.40±1.03	27.67±0.99	25.38±02.02	34.17±18.24	29.74±6.31
CD4/mm^3^	1224.33±280.17	980.05±85.65	1,010.84±82.62	501.41±20.20	529.64±205.56	515.42±45.79 *
VL (c/ml)	588,773.27±219,049.43	98,397.45±29,810.18	177,722.95±45,527.78 §	87,847.41±7,816.82	67,548.55±9,404.65	77,776.66±7,782.99 *

§ indicates significant differences between naïve and treated individuals within either pediatric or adult HIV-1B populations.

* indicates significant differences between pediatric and adult populations for total values.

The pediatric and adult cohorts differed in average CD4/mm^3^ and viral load, but not in % CD4 (Table 2). Thus, we further analysed whether the relative importance of CD4 cells count (both %CD4 and CD4/mm^3^), VL (c/ml), children age, and of ART experience, also differed in the pediatric and adult cohorts of patients by including these variables in a Principal Component Analysis (PCA) (Table 3). In the pediatric cohort, such PCA yielded three main Principal Components (PCs) that collectively explained 84% of the total variance. Children age was highly associated with PC1, ART experience with PC2 and CD4/mm^3^ with PC3 (Squared loadings ≥ 91.2%). In the adult cohort, PCs 1, 2 and 3 were associated with ART experience, VL and % CD4, respectively, age being associated with the less important PC (Table 3). Thus, the relative importance of the five clinical parameters analysed also differed between the pediatric and the adult cohorts.

**Table 3:** Principal component analysis of five clinical factors from 163 pediatric and adult HIV-1B infected patients of the Madrid cohort.

	Pediatric (age 0–21)						Adult (age >21)				
Principal Component	1	2	3	4	5	Principal Component	1	2	3	4	5	
***Percent association component-variable***												
Age	**91.2**	1.9	0.3	0.1	7.5	ART experience	**95.8**	0.1	0.0	4.0	0.1	
ART experience	0.0	**93.7**	2.3	3.9	0.1	VL	0.1	**95.6**	1.2	2.9	0.2	
CD4/mm^3^	1.0	2.2	**94.7**	2.0	0.1	% CD4	0.0	1.2	**96.4**	2.4	0.0	
VL	0.3	5.4	2.7	**84.6**	7.0	CD4/mm^3^	5.2	3.7	3.1	**87.9**	0.1	
% CD4	11.6	0.1	0.2	8.4	**79.7**	Age	0.2	5.2	6.5	3.1	**85.0**	
***Expected values under broken-stick model***	20.8	20.3	20.2	19.8	18.9		20.3	21.2	21.4	20.0	17.1	
***Total variance explained by the component***	40.7	30.0	13.6	10.5	5.2		44.8	25.4	18.8	5.9	5.1	

Given that the HIV-1B between-host evolution and the relative importance of the analysed clinical factors differed in pediatric and adult cohorts, we studied how these clinical factors affected the between-host evolution of the pediatric HIV-1B population, and we used the adult-infecting virus population for comparison purposes.

### Relative contribution of clinical factors to the between-host evolution of pediatric HIV-1B populations

We explored the relative contribution of five clinical factors to the evolution of the pediatric HIV-1B population. To do so, we estimated *d*, *d*_N_, *d*_S_ and *d*_N_/*d*_S_ for each sequence in the pediatric HIV-1B population by averaging values of each evolutionary parameter derived from pairwise comparisons with the rest of the sequences in the data set. For each evolutionary parameter we fit a global model containing all the clinical factors, and a set of nested models containing all possible combinations of the clinical factors, and we calculated the AIC for each model. The model with the lowest AIC (best-ranked model) was considered as the best in predicting values of each evolutionary parameter (Table 4). The best-ranked model for *d*, *d*_N_ and *d*_N_/*d*_S_ in the HIV-1B pediatric population included the clinical factors children age and ART experience, which explained more than half of the variation in these evolutionary parameters (*R*^*2*^>0.55). The models including CD4/mm^3^ and children age, ART experience and CD4/mm^3^, or combining CD4/mm^3^, ART experience and children age closely competed with the best-ranked model (Δ_*i*_<4; *R*^2^>0.47). However, *ω*_*i*_ for these models was two- to three-fold smaller than for the best-ranked model (Table 4), indicating that the best-ranked model was significantly better than models with Δ_*i*_<4. The rest of the models poorly predicted *d*, *d*_N_ and *d*_N_/*d*_S_ (Δ_*i*_ > 4; *R*^2^<0.30). Ambiguous results were obtained for *d*_S_, as up to ten models closely competed with the best-ranked model, and none of them explained more than 37% of the variance in *d*_S_ (Table 4). Thus, children age, ART experience and CD4/mm^3^, which explained the majority of the variance in the HIV-1B-infected pediatric cohort of patients, were also the best predictors of virus evolution. We therefore analysed in more detail the effect of these three clinical factors in the evolution of the pediatric HIV-1B population.

**Table 4:** Model selection analyses for genetic diversity (*d*), rate of synonymous (*d*_*S*_) and non-synonymous substitutions, and selection pressures (*d*_*N*_*/d*_*S*_). Model structures included children age (Age), ART experience (ART), CD4 count (both as % CD4 and as CD4/mm^3^), and viral load (VL). Best-ranked models are bolded and have the lowest AIC value.

Model structure^1^	*R*^2^	logLik	AIC^2^	Δ_i_^3^	*ω*_*i*_^4^
***d***					
***ART (61%) + Age (39%)***	**0.55**	**315.05**	**-620.90**	**0**	**0.335**
*ART (80%)+ CD4/mm*^*3*^*(20%)*	0.55	315.05	-618.11	2.79	0.083
*ART (55%) + Age (35%) + CD4/mm*^*3*^ *(10%)*	0.55	315.05	-618.10	2.80	0.054
*Age (71%) + CD4/mm*^*3*^*(29%)*	0.47	312.62	-617.23	3.67	0.054
***d***_***N***_					
***ART (61%) + Age (39%)***	**0.66**	**337.21**	**-664.91**	**0**	**0.364**
*ART (61%)+ CD4/mm*^*3*^*(39%)*	0.61	337.40	-662.79	2.12	0.126
*ART (54%) + Age (32%) + CD4/mm*^*3*^ *(14%)*	0.61	337.40	-662.78	2.13	0.125
*Age (58%) + CD4/mm*^*3*^*(42%)*	0.57	335.58	-661.16	3.75	0.056
***d***_***S***_					
***VL (100%)***	**0.37**	**242.07**	**-476.94**	**0**	**0.195**
*CD4/mm*^*3*^*(100%)*	0.29	241.46	-474.93	2.01	0.071
*VL (75%) + %CD4 (25%)*	0.38	242.28	-474.55	2.39	0.059
*Age (100%)*	0.25	241.26	-474.53	2.41	0.058
*VL (92%) + ART (8%)*	0.38	242.26	-474.52	2.42	0.058
*VL (89%) + Age (11%)*	0.37	242.09	-474.17	2.77	0.049
*VL (99%) + CD4/mm*^*3*^ *(1%)*	0.37	242.07	-474.15	2.79	0.048
*ART (100%)*	0.10	241.05	-474.09	2.85	0.047
*%CD4 (100%)*	0.07	241.04	-474.08	2.86	0.047
*Age (64%) + %CD4 (36%)*	0.34	241.77	-473.54	3.40	0.036
*CD4/mm*^*3*^ *(80%) + %CD4 (20%)*	0.33	241.74	-473.48	3.46	0.035
***d***_***N***_***/d***_***S***_					
***ART (73%) + Age (27%)***	**0.57**	**48.38**	**-86.96**	**0**	**0.274**
*ART (63%)+ CD4/mm*^*3*^*(37%)*	0.57	48.51	-85.03	1.93	0.104
*ART (56%) + Age (31%) + CD4/mm*^*3*^ *(14%)*	0.57	48.49	-84.98	1.98	0.102
*Age (58%) + CD4/mm*^*3*^*(42%)*	0.54	47.12	-84.24	2.73	0.070
*ART (51%) + Age (31%) + CD4/mm*^*3*^*(12%) + VL (6%)*	0.58	48.91	-83.82	3.14	0.057

^1^ Identity of clinical factors and their corresponding relative importance in each predictive model of each HIV-1B evolutionary parameter.

^2^AIC, Akaike’s Information Criterion.

^3^ Where *i* = the model in question and 0 = best-ranked model.

^4^ AIC model weight; the larger the *ω*, the greater the likelihood of the model given the data, relatively to the competing models [51].

### Association of children age with the evolution of the pediatric HIV-1B population

**Fig 2:**
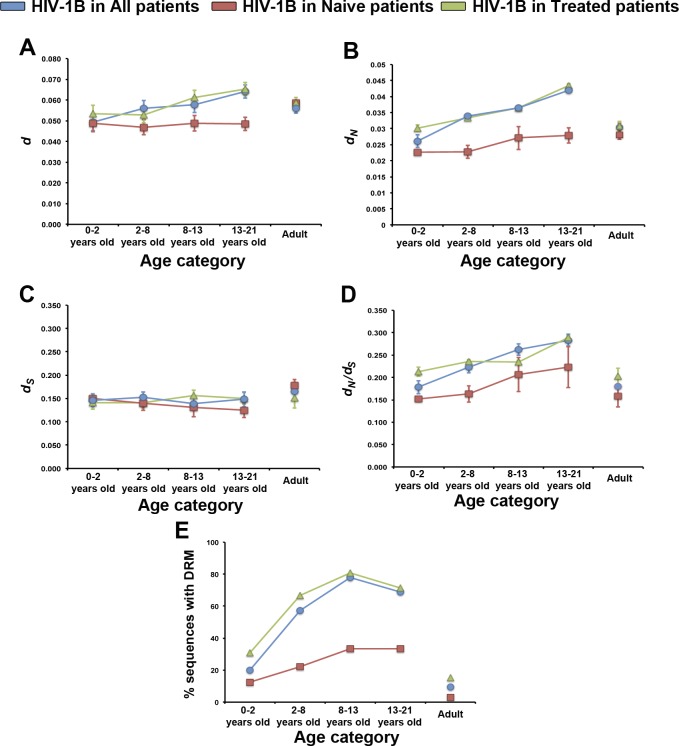
Association between HIV-1B evolution and children age. HIV-1B genetic diversity (A), rate of non-synonymous (B) and synonymous (C) substitutions, selection pressures (D), and frequency of sequences with DRMs (E) in the pediatric HIV-1B population across children age categories. Data of the corresponding adult-infecting HIV-1B populations is also presented. Values in naïve patients (red squares), treated patients (green triangles), and grouping both classes of patients (blue circles) are shown. Values indicate mean±standard error. Note the different scale in each panel.

To analyse the effect of children age in the between-host evolution of HIV-1B, we divided the pediatric cohort into four age categories following the CDC recommendations, so that our results could be clinically meaningful [53]: 0–2 years old, 2–8 years old, 8–13 years old and 13–21 years old, with between 30 and 50 sequences each. We also included the adult cohort as an additional age category (Fig 2). To control for sample size effects we created ten balanced datasets with 30 (smallest sample size across children age categories) randomly-sampled HIV-1B sequences from the adult-infecting 163-sequence datasets, and evolutionary parameters in these balanced datasets did not significantly differ from those in the real data (S2 Table). Thus, we kept the 163-sequence datasets for consistency with our previous analyses.

HIV-1B population genetic diversity increased with children age (from 0.049±0.004 to 0.064±0.003; *F* = 166.97, *P*<1x10^-5^) (Fig 2A). Interestingly, this increase in HIV-1B genetic diversity with children age was primarily due to an increase in the rate of non-synonymous substitutions (*d*_N_: from 0.026±0.002 to 0.042±0.001; *F* = 311.22, *P*<1x10^-5^) (Fig 2B), whereas the rate of synonymous substitutions remained similar across age categories (*d*_S_: 0.147±0.001 to 0.148±0.001; *F* = 0.52, *P* = 0.471) (Fig 2C). Consequently, purifying selection was weaker at increasing children age (*d*_N_/*d*_S_: from 0.178±0.001 to 0.283±0.003; *F* = 269.77, *P*<1x10^-5^) (Fig 2D), although the frequency of sites under purifying and diversifying selection did not change across age categories (67/334 to 79/334 and 7/334 to 11/334, respectively; *χ*^*2*^<1.01, *P*>0.798). In the adult virus population, *d* and *d*_N_ were smaller than in children over 2 years old (Fig 2A and 2B), and *d*_S_ was higher than in every children age category (Fig 2C). Thus, purifying selection in the adult HIV-1B population was stronger than in every children age category (Fig 2D).

To analyse whether the changes in the rate of non-synonymous substitutions across children age categories were associated with HIV-1B drug resistance, we determined the number of virus sequences harbouring DRMs across children age categories (Fig 2E). Such frequency was higher at increasing children age (from 23% to 69%; *χ*^*2*^ = 6.84, *P*<0.009), and always higher than in the adult HIV-1B population (10%) (Fig 2E). The proportion of pediatric HIV-1B sequences harbouring mutations conferring resistance to nucleoside reverse transcriptase and protease inhibitors (NRTI and PI, respectively) significantly increased across age categories (*χ*^*2*^>4.49, *P*<0.034): from 13% and 20% in 0–2 years old patients, to 67% and 38% in 13–21 years old patients, respectively. The proportions of HIV-1B sequences harbouring mutations conferring resistance to non-nucleoside reverse transcriptase inhibitors (NNRTI) also increased across age categories (from 23% to 48%), but this increase was not significant (*χ*^*2*^ = 1.63, *P* = 0.202) (S3 Table). The frequency of drug resistance-associated sites under purifying and diversifying selection did not change across age categories (7/47 to 10/47 and 0/47 to 3/47, respectively; *χ*^*2*^<4.77, *P*>0.261).

### Association of ART experience with the evolution of the pediatric HIV-1B population

We compared *d*, *d*_N_, *d*_S_ and *d*_N_/*d*_S_ in virus populations infecting naïve (n = 30) and treated children (n = 133), including in the analyses the corresponding adult-infecting HIV-1B population (Table 2). Again, we controlled for sample size effects by creating ten balanced datasets with 30 (number of naïve HIV-1B pediatric sequences in our dataset) randomly sampled HIV-1B sequences from treated pediatric patients. Evolutionary parameters in these balanced datasets did not significantly differ from those in the real data (S4 Table), such that we kept data from the 133-treated sequences dataset.

In pediatric patients, HIV-1B genetic diversity was higher in treated than in naïve pediatric patients (0.061±0.002 *vs*. 0.047±0.002; *F* = 268.34, *P*<1x10^-5^). This was due to a nearly 2-fold difference in the rate of non-synonymous substitutions (*d*_*N*_: 0.039±0.000 *vs*. 0.023±0.000; *F* = 513.69, *P*<1x10^-5^), whereas no significant differences in the rate of synonymous substitutions were observed (*d*_*S*_: 0.143±0.002 *vs*. 0.147±0.000; *F* = 1.98, *P* = 0.260). Accordingly, purifying selection was weaker in the treated than in the naïve HIV-1B pediatric population (*d*_*N*_/*d*_*S*_: 0.262±0.001 *vs*. *0*.163±0.003; *F* = 429.02, *P*<1x10^-5^) (Table 2). The frequency of sites under purifying selection was lower in naïve than in treated virus populations (26/334 *vs*. 137/334; *χ*^*2*^ = 105.81, *P*<1x10^-5^), with no changes in the frequency of positively selected sites (7/334 *vs*. 11/334; *χ*^*2*^ = 0.91, *P* = 0.339). In adult patients, HIV-1B genetic diversity and the rate of non-synonymous substitutions did not differ between naïve and treated virus populations (*d*: 0.059±0.003 *vs*. 0.056±0.003, *d*_*N*_: 0.028±0.002 *vs*. 0.031±0.003; *F*<1.03, *P*>0.126). However, the rate of synonymous substitutions was higher in the HIV-1B naïve population (*d*_*S*_: 0.178±0.003 *vs*. 0.152±0.002; *F* = 735.72, *P*<1x10^-5^), due to stronger purifying selection (*d*_*N*_/*d*_*S*_: 0.158±0.002 *vs*. 0.203±0.002; *F* = 947.16, *P*<1x10^-5^) (Table 2). The frequency of sites under purifying and under diversifying selection did not vary between naïve and treated virus populations (227/334 *vs*. 216/334 and 16/334 *vs*. 18/334, respectively; *χ*^*2*^<0.17, *P*>0.680). Interestingly, although *d*, *d*_*N*_ and *d*_*S*_ were higher in naïve adult than in naïve pediatric HIV-1B populations, the opposite trend was observed in treated patients (Table 2).

**Fig 3:**
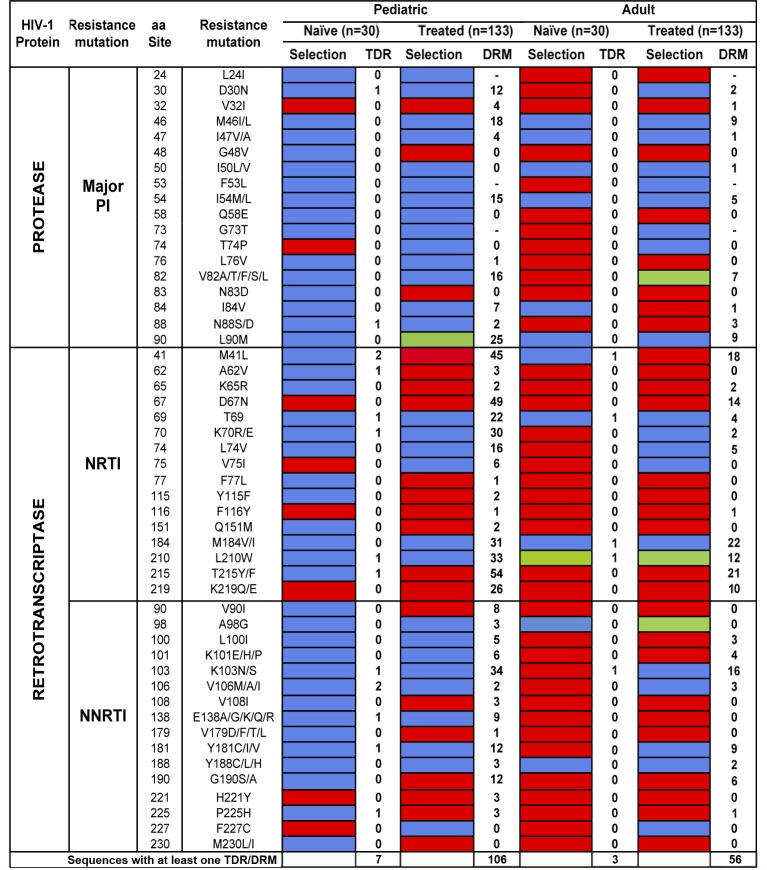
Selection pressures and prevalence of mutations in amino acid residues associated with antiretroviral drug resistance. Selection pressures in amino acid residues associated with drug resistance in pediatric and adult HIV-infected patients according to their ART experience. Sites under diversifying selection are in green; sites under purifying selection are in red. Sites under neutral evolution are in blue. DRM prevalence was determined following the WHO TRM list for ‘naïve’ patients [45], and the IAS–USA 2014 list for ‘treated’ patients [47]. PI: protease inhibitors; NRTI: nucleoside reverse transcriptase inhibitors; NNRT: non-NRTI; TDR: transmitted drug resistance; DRM: drug resistance mutations. Dashes: not a major mutation in treated patients.

In pediatric patients, the proportion of sequences harbouring at least one DRM was significantly higher in the treated than in the naïve virus population (106/133 *vs*. 7/30, *χ*^*2*^ = 35.36, *P*<1x10^-4^). The same trend was observed when the frequency of PI, NRTI and NNRTI mutations was analysed separately (S3 Table). In the naïve HIV-1B pediatric population, only 17% of the sites associated with drug resistance were under purifying selection; while in the treated population 43% of the sites were under purifying selection (8/47 *vs*. 20/47; *χ*^*2*^ = 7.32, *P* = 7x10^-3^) (Fig 3). Only one site was found to be under diversifying selection in HIV-1B populations of treated patients and none in virus populations of naïve pediatric patients (Fig 3). In HIV-1B infected adults, the proportion of sequences harbouring at least one DRM was again higher in treated than in naïve virus populations (56/133 *vs*. 3/30, *χ*^*2*^ = 10.93, *P* = 1x10^-3^), with the same trends for PI, NRTI and NNRTI mutations (S3 Table). In naïve adult patients, 80% of the sites associated with drug resistance were under purifying selection; while in the treated population around 60% of the sites were under purifying selection (38/47 *vs*. 28/47; *χ*^*2*^ = 5.09, *P* = 0.024) (Fig 3). No differences in the frequency of sites under diversifying selection among HIV-1B populations in adults were observed (1/47 vs. 3/47; *χ*^*2*^>0.11, *P*<0.617). When pediatric and adult HIV-1B populations were compared, the proportion of sequences harbouring DRMs was higher in the pediatric than in the adult virus population for both treated and naïve patients (*χ*^*2*^>2.29, *P*<0.049). The number of drug resistance-associated sites under purifying selection was higher in adult than in pediatric virus populations both in naïve and in treated patients (*χ*^*2*^>5.19, *P*<0.019), this difference being much larger in naïve patients (Fig 3).

### Association of children CD4 count with the evolution of the pediatric HIV-1B population

**Fig 4:**
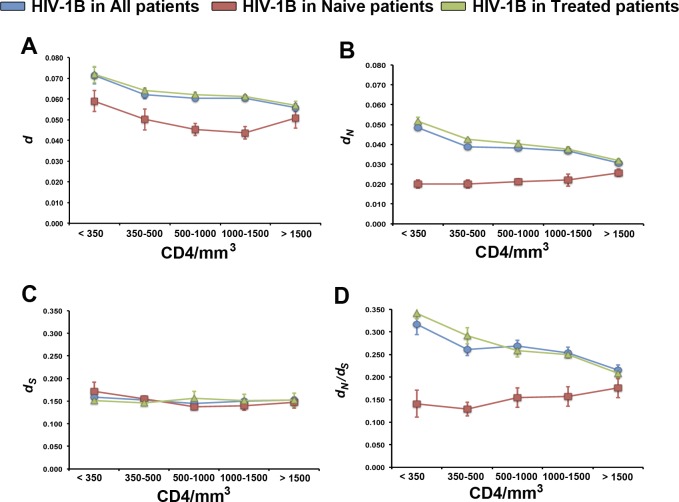
Association between HIV-1B evolution and CD4 cells count. HIV-1B genetic diversity (A), rate of non-synonymous (B) and synonymous (C) substitutions, and selection pressures (D) in the pediatric HIV-1B population across CD4/mm^3^ categories. Values in naïve patients (red squares), treated patients (green triangles), and grouping both classes of patients (blue circles) are shown. Values indicate mean±standard error. Note the different scale in each panel.

To analyse the role of the concentration of CD4 cells (CD4/mm^3^) in HIV-1B evolution, we divided both the pediatric and adult cohorts into five categories as defined by the CDC classification [53]: >1500 CD4/mm^3^, 1500–1000 CD4/mm^3^, 1000–500 CD4/mm^3^, 500–350 CD4/mm^3^ and <350 CD4/mm^3^, with between 12 and 57 sequences each, and we estimated the same evolutionary parameters as above (Fig 4 and S1 Fig). In the pediatric HIV-1B population, genetic diversity decreased with increased CD4/mm^3^ (*d*: from 0.071±0.004 to 0.056±0.002; *F* = 62.52, *P*<1x10^-5^) (Fig 4A). This decrease of HIV-1B genetic diversity was due to a reduction of *d*_N_ (*d*_N_: from 0.048±0.001 to 0.031±0.000; *F* = 102.60, *P*<1x10^-5^) (Fig 4B), while *d*_S_ remained constant across CD4/mm^3^ categories (*d*_S_: from 0.159±0.001 to 0.150±0.001; *F* = 1.56, *P* = 0.213) (Fig 4C). Accordingly, purifying selection was weaker at lower CD4 count (*d*_N_/*d*_S_: from 0.317±0.003 to 0.215±0.002; *F* = 65.91, *P*<1x10^-5^) (Fig 4D). The frequency of sites under purifying and diversifying selection did not vary across CD4/mm^3^ categories (8/334 to 20/334 and 1/334 to 6/334, respectively; *χ*^*2*^<0.49, *P*>0.493). In the adult HIV-1B population, HIB-1B genetic diversity (*d*: 0.052±0.002–0.065±0.006; *F*<1.53, *P*>0.217) did not significantly change with increasing CD4/mm^3^ (S1 Fig). Similarly, both *d*_N_ and *d*_S_ did not vary at increasing CD4/mm^3^ (*d*_N_: 0.024±0.001–0.032±0.000; *d*_S_: 0.155±0.005–0.195±0.010; *F*<2.58, *P*>0.108). Finally, neither selection pressures (*d*_N_/*d*_S_: 0.147±0.012–0.174±0.002; *F* = 3.38, *P* = 0.423), nor the frequency of sites under purifying and diversifying selection (84/334 to 95/334 and 1/334 to 4/334, respectively; *χ*^*2*^<0.53, *P*>0.527) did vary across CD4/mm^3^ categories.

### Interactive effects of clinical factors in the evolution of the pediatric HIV-1B population

Because the best-ranked models explaining HIV-1B evolution in pediatric populations were those including ART+Age and ART+CD4/mm^3^ (Table 4), we focused in these two interactions. We used the HIV-1B sequence datasets from naïve and treated patients, and in each of them we explored how *d*, *d*_N_, *d*_S_ and *d*_N_/*d*_S_ varied both across age and across CD4 count categories (Figs 2 and 4).

HIV-1B genetic diversity in naïve pediatric patients remained constant across age categories (*d*: 0.047±0.004–0.049 ±0.003; *F* = 0.30, *P* = 0.824). Similar results were obtained when *d*_N_ (0.023±0.001–0.028±0.002; *F* = 1.05, *P* = 0.373) and *d*_S_ (0.125±0.016–0.150±0.004; *F* = 0.95, *P* = 0.417) were analysed separately. Accordingly, selection pressures did not vary with children age (*d*_N_/*d*_S_: from 0.151±0.006 to 0.223±0.046; *F* = 2.01, *P* = 0.115) (Fig 2). In addition, the proportion of virus sequences harbouring NRTI and NNRTI mutations increased from 13% to 33%, and the proportion of sequences with PI mutations from 0% to 33%. However, these increases were not significant (*χ*^*2*^<0.74, *P*>0.390) (S3 Table). On the other hand, in treated patients virus *d* increased with age (*d*: from 0.053±0.004 to 0.065±0.003; *F* = 81.71, *P*<1x10^-5^), which was due to a smaller increase in *d*_S_ (*d*_S_: 0.141±0.001–0.156±0.002; *F* = 63.14, *P*<1x10^-5^), and to a larger increase in *d*_N_ (*d*_N_: 0.030±0.001–0.043±0.001; *F* = 86.92, *P*<1x10^-5^). Consequently, purifying selection was weaker with increasing children age (*d*_N_/*d*_S_: from 0.213±0.010 to 0.289±0.003; *F* = 25.92, *P*<1x10^-5^) (Fig 2). The proportion of HIV-1B sequences harbouring NRTI mutations significantly increased across age categories, from 31% in 0–2 years old patients to 71% in 13–21 years old patients (*χ*^*2*^ = 5.26, *P*>0.022). The proportions of HIV-1B sequences harbouring NNRTI and PI mutations also increased across age categories (from 39% to 55% for NNRTI mutations, and from 31% to 41% for PI mutations), but this increase was not significant (*χ*^*2*^<0.74, *P*>0.390) (S3 Table). Values of all the analysed evolutionary parameters were generally higher in the HIV-1B population of treated than of naïve children (Fig 2).

Analyses in HIV-1B naïve pediatric populations across CD4/mm^3^ categories indicated that genetic diversity (*d*: 0.059±0.005–0.051±0.005), the rate of synonymous (*d*_S_: 0.138±0.007–0.172±0.020) and non-synonymous (*d*_N_: 0.020±0.002–0.026±0.002) substitutions, and selection pressures (*d*_N_/*d*_S_: 0.129±0.015–0.175±0.021) did not depend on CD4/mm^3^ values (*F*<0.93, *P*>0.460) (Fig 4). In treated children, HIV-1B genetic diversity decreased at larger CD4/mm^3^ values (*d*: 0.072±0.004 to 0.057±0.002; *F* = 18.30, *P*<1x10^-5^). This was due to a decrease of *d*_N_ (*d*_N_: 0.052±0.002 to 0.032±0.001; *F* = 18.30, *P*<1x10^-5^), whereas *d*_S_ did not vary across CD4/mm^3^ categories (*d*_S_: 0.149±0.005–0.153±0.005; *F* = 2.03, *P* = 0.088). Hence, purifying selection in the HIV-1B population was stronger with increasing CD4/mm^3^ (*d*_N_/*d*_S_: from 0.346±0.013 to 0.208±0.008; *F* = 20.25, *P*<1x10^-5^). Values of all the analysed evolutionary parameters were higher in the HIV-1B population of treated than of naïve children, with the exception of *d*_S_ (Fig 4).

Thus, the observed greater HIV-1B genetic diversity with increased children age and reduced CD4 count can be mainly attributed to virus evolution in treated pediatric patients, and specifically to an increase in the rate of non-synonymous substitutions. Moreover, such increase is accompanied by a higher frequency of drug resistance mutations.

## Discussion

It is currently well-know that the course of HIV infection and the virus epidemiological dynamics differ in pediatric and adult patients [4,22,24,25,28]. Given such differences, it might seem obvious that virus evolution would be different in children and adults. However, to date there is little experimental evidence of such differences. We performed a detailed analysis of HIV-1B evolution in comparable adult and pediatric cohorts that differ in clinical and epidemiological traits. Indeed, patients of our study adult cohort had on average lower concentration of susceptible cells and reduced levels of HIV-1 replication as compared with those of the pediatric cohort. Moreover, both cohorts also differed in relevant traits for virus epidemiology: Transmission rates were higher in the adult cohort than in the pediatric cohort of Madrid [35,37]; and the primary mode of HIV-1B transmission was also different, as the pediatric cohort only contained patients infected by mother-to-child transmission. Such variation in clinical and epidemiological factors was accompanied by differences in HIV-1B evolution in children and adults: Genetic diversity was higher in adult- that in children-infecting HIV-1B populations, due to a higher rate of synonymous substitutions in the adult HIV-1B population. Accordingly, purifying selection was weaker in viruses infecting pediatric patients than in adults. Mathematical models on the evolution of RNA viruses (like HIV-1) predict that, in populations with different epidemiological dynamics, factors affecting within-host virus evolution such as decrease of susceptible cells and lower viral replication rates, would accelerate between-host virus evolution [7,8]. This would explain the higher genetic diversity in adult-infecting HIV-1B populations. Two conditions need to be met to establish this link between within- and between-host evolution: First, host age of infection (i.e. exposure time to the virus *sensu* [54]) upon transmission must be different in the two host populations. In our study, there is a marked difference in the age of infection as all patients from the pediatric cohort were perinatally infected, whereas this was not so in the adult cohort. Second, within-host evolutionary rates must change over the course of infection. Here, we have not measured within-host evolutionary rates, as we did not count with sequential samples of the same patient. However, indicatives of lower within-host virus evolutionary rates, such as depletion of target CD4 cells as well as changes in viral load during the course of the HIV-1B infection [8], have been reported for both the adult and the pediatric cohorts of Madrid [37,38]. Thus, the analysed populations meet the conditions to establish an association between within- and between-host evolution. Indeed, our results would be also in agreement with experimental analyses in other RNA viruses that explained changes in evolutionary rates across virus populations as a function of contact rates, replication rates, speed of depletion of susceptible individuals and/or the mode of transmission [8,10–12].

The observed differences in HIV-1B evolution between children and adults suggested that the relative importance of the factors driving virus evolution might differ in these two virus populations. However, experimental analyses of the clinical factors driving HIV-1 evolution are largely restricted to adult patients. Thus, we focused in the pediatric HIV-1B population. The factors detected by our PCA analyses as defining traits of the pediatric cohort (ART experience, children age and CD4/mm^3^, which together explained 85% of the total variance in the pediatric cohort) had the greatest impact on the variance in HIV-1B *d*, *d*_*N*_, and *d*_N_/*d*_S_. The CD4/mm^3^ had the lesser impact in these evolutionary parameters, this factor being included only in some of the best-raked models and having lower relative importance. In contrast, viral load and percentage of CD4 cells did not appear to contribute meaningfully to HIV-1B evolution in children. Importantly, the best-ranked models explained 55–66% of the variance in the HIV-1B evolutionary parameters (except for *d*_S_). Such values are evidence that the combined effect of the clinical factors considered in these models significantly determine between-host HIV-1B evolution, and with different relative importance. Accordingly, previous works attributed a role to ART experience, children age and CD4 count as determinants of within-host HIV-1 evolution of adult and children cohorts when considered individually [3,17,18,55,56]. Still, in our models part of the variance in HIV-1B between-host evolutionary parameters remained unexplained, which indicate that clinical factors other than those considered here have an effect in the evolution of pediatric HIV-1B populations. For instance, due to lack of information in the analysed cohort we have not considered the type/number of ARTs undergone by each patient, or adherence to treatment, which have been proposed to partially determine HIV-1B evolution [21,25,36]. The lack of such information would potentially blur our model selection analysis if only certain drug combinations would affect virus evolution, which would therefore yielded a poor predictive power of ART experience in HIV-1B evolution in our analyses. However, our approach was robust enough to detect ART experience as a major factor in virus evolution. In any case, future work that will add these factors as predictors would contribute to a finer mapping of HIV-1B evolution determinants.

We further explored how the most relevant clinical factors for HIV-1B evolution modulated virus between-host evolution in pediatric patients. Our results indicate that HIV-1B genetic diversity increases with children age. It is important to note that the pediatric patients included in our analyses were perinatally infected, so that in our study population children age and age of infection are largely equivalent. However, both have been shown to similarly affect HIV-1B evolution [3,17]. In perinatally infected children, lower HIV-1 genetic diversity at younger age (or early stages of infection) has been associated with genetic drift [57] due to severe bottlenecks during mother-to-child transmission (e.g. [58,59]), but also with selection of HIV-1 variants adapted to vertical transmission (reviewed by [60]). Our results provide some insights on which of these two phenomena may determine the genetic diversity of the analysed HIV-1B population in younger children. If genetic drift were the major driver, we would expect that the HIV-1B population in children under 2 year-old were a random representation of that in the equivalent adult population, and therefore with similar genetic diversity [61]. However, genetic diversity in children from 0 to 2 year-old was significantly lower than in adults, which suggests that certain selection is acting on the virus variants that are vertically transmitted. After perinatal transmission, very high rates of HIV-1 replication have been detected in younger infected children that can persist for long periods of time, especially in untreated patients [25,62]. High rates of virus replication increase HIV-1B population sizes and, due to the error-prone nature of the HIV-1B polymerase, may result in random accumulation of mutations in the virus population. Such accumulation would increase the genetic diversity of the virus population with children age/age of infection, which would explain our results. However, two of our observations argue against this possibility. First, viral load was a poor predictor of virus genetic diversity; hence within-host population size is not a factor in determining between-host evolution of HIV-1B in pediatric patients of the Madrid cohort. Second, larger virus population sizes are often associated with increasing rates of synonymous mutations [2,9], but we observed that higher HIV-1B genetic diversity was rather accompanied by higher rates of non-synonymous mutations. Therefore, the observed changes in virus genetic diversity across age categories are likely the consequence of adaptive changes, rather than of neutral evolution.

The most obvious forces shaping HIV-1B adaptive evolution may be host- and/or environmental-related selection pressures; i.e., the strength of the host immune system and/or antiviral drugs, respectively. These two types of selection pressures are linked to two of the clinical factors detected as predictors of HIV-1B evolution: CD4 count and ART experience. At early stages of infection, the children immune system is still immature, exerting a smaller selection pressure on the virus population [22,23]. With increasing children age, the virus is subjected to stronger selection pressures to overcome the immune system [3,63]. If this were the major mechanism shaping the evolution of the pediatric HIV-1B populations from Madrid, the analysis of virus evolutionary parameters in the entire virus pediatric population would mimic the observations in naïve children. However, analyses of changes in evolutionary parameters of pediatric virus populations across both age and CD4 count categories indicated that such changes were associated with HIV-1B evolution in ART experienced, but not in naïve, children. Moreover, the interaction between children age and ART experience, and of CD4 count and ART experience, were the best predictors of virus population *d*, *d*_N_, and *d*_N_/*d*_S_, ART experience having always the highest relative importance. These results indicate that the roles of children age/age of infection and of CD4 count in the pediatric HIV-1B evolution are likely linked to the effects of ART, and highlight the importance of considering the combined effect of different clinical factors to better understand HIV-1 evolution. Our findings are at odds with previous analyses that failed in finding a significant interaction between children age/age of infection and ART experience in determining the genetic diversity of pediatric HIV-1B populations [3]. However, such work considered children between 0 and 4 year-old, which might not be enough time span to detect the ART x Age interaction. Indeed, some of the trends observed in our study population would disappear if we would restrict our analyses to the youngest age categories (Fig 2).

Altogether, our results strongly suggest that ART experience is a major determinant of pediatric HIV-1B genetic diversity. Hence, we would expect that virus evolution would favour the fixation of mutations conferring ART resistance. In our study population, the frequency of DRMs in the HIV-1B pediatric population increased with children age, in parallel with changes in the virus genetic diversity and the rate of non-synonymous substitutions, in particular in ART experience children. This is compatible with the central role of ART experience in pediatric HIV-1B evolution, and suggests that the same factors affecting HIV-1B evolution influence the fixation of DRMs. Thus, the genetic diversity of pediatric HIV-1B populations could be a good predictor of the virus ability to generate DRMs, and our results may also serve as scaffolding for building mathematical models aiming at predicting the conditions that favour the appearance of DRMs in children.

## Supporting Information

S1 Fig Association between HIV-1B evolution and CD4/mm^3^ in adult patients. HIV-1B genetic diversity (A), rate of non-synonymous (B) and of synonymous (C) mutations, and selection pressures (D) in the adult-infecting HIV-1B population across CD4/mm^3^ categories. Red squares indicate values in naïve patients, green triangles indicate values in treated patients, and blue circles indicate values groping both classes of patients. Values indicate mean±standard error. Note the different scale in each panel.
(TIF)

S1 File Information clinical and HIV-1B sequence data of patients enrolled in the study. Data on age, CD4 counts (percentage and cells/mm^3^), viral load (HIV-1B-RNA copies/ml of plasma, c/ml), presence of drug resistance mutations at the moment of HIV-1B sampling, and accession number of virus sequences.
(XLSX)

S1 Table Estimates of evolutionary parameters for the ten “balanced” (n = 163) and the complete (n = 450) partial *pol* datasets from the adult-infecting HIV-1B population. (DOCX)

S2 Table Estimates of evolutionary parameters for the ten “balanced” (n = 30) and the complete (n = 163) partial *pol* datasets from the adult-infecting HIV-1B population. (DOCX)

S3 Table Frequency (%) of sequences with at least one drug resistance mutation in the pediatric HIV-1B population according to patient age. (DOCX)

S4 Table Estimates of evolutionary parameters for the ten “balanced” replicates (n = 30) and the complete (n = 133) partial *pol* datasets from the HIV-1B population infecting treated children. (DOCX)
