# Supplementary figures and images for: Clinical Determinants of HIV-1B Between-Host Evolution and their Association with Drug Resistance in Pediatric Patients
Source: PLoS One. 2016 Dec 1;11(12):e0167383. doi: 10.1371/journal.pone.0167383 (PMC5132210; doi:10.1371/journal.pone.0167383)

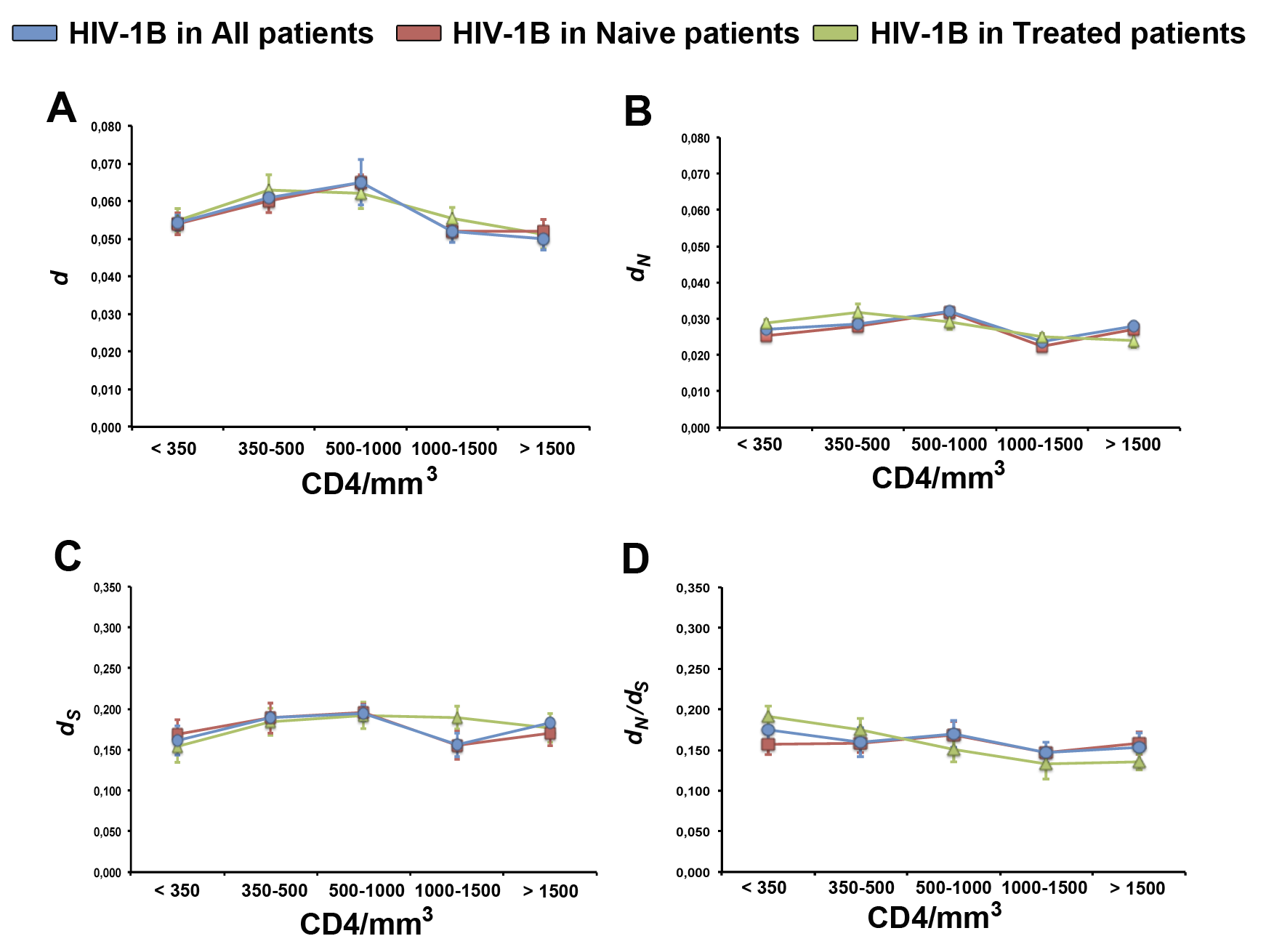

Supplement: S1 Fig — HIV-1B genetic diversity (A), rate of non-synonymous (B) and of synonymous (C) mutations, and selection pressures (D) in the adult-infecting HIV-1B population across CD4/mm3 categories. Red squares indicate values in naïve patients, green triangles indicate values in treated patients, and blue circles indicate values groping both classes of patients. Values indicate mean±standard error. Note the different scale in each panel. (TIF) [file pone.0167383.s001.tif]
