# Supplementary material for: Clinical Determinants of HIV-1B Between-Host Evolution and their Association with Drug Resistance in Pediatric Patients
Source: PLoS One. 2016 Dec 1;11(12):e0167383. doi: 10.1371/journal.pone.0167383 (PMC5132210; doi:10.1371/journal.pone.0167383)
Supplement: S2 Table — (DOCX) [file pone.0167383.s004.docx]

**S2 Table.** Estimates of evolutionary parameters for the ten “balanced” (n=30) and the complete (n=163) partial *pol* datasets from the adult-infecting HIV-1B population.

|  |  |  | ***d*** |  | ***d_N_*** |  | ***d_S_*** |  | ***d_N_/d_S_*** |
| --- | --- | --- | --- | --- | --- | --- | --- | --- | --- |
| Balanced 1 |  |  | 0.056±0.002 |  | 0.031±0.000 |  | 0.162±0.002 |  | 0.181±0.002 |
| Balanced 2 |  |  | 0.057±0.002 |  | 0.031±0.000 |  | 0.166±0.002 |  | 0.187±0.002 |
| Balanced 3 |  |  | 0.059±0.002 |  | 0.030±0.000 |  | 0.164±0.002 |  | 0.183±0.002 |
| Balanced 4 |  |  | 0.055±0.002 |  | 0.029±0.000 |  | 0.160±0.002 |  | 0.181±0.002 |
| Balanced 5 |  |  | 0.058±0.002 |  | 0.030±0.000 |  | 0.164±0.002 |  | 0.183±0.002 |
| Balanced 6 |  |  | 0.057±0.002 |  | 0.032±0.000 |  | 0.166±0.002 |  | 0.183±0.002 |
| Balanced 7 |  |  | 0.058±0.002 |  | 0.031±0.000 |  | 0.163±0.002 |  | 0.180±0.002 |
| Balanced 8 |  |  | 0.058±0.002 |  | 0.030±0.000 |  | 0.165±0.002 |  | 0.180±0.002 |
| Balanced 9 |  |  | 0.056±0.002 |  | 0.031±0.000 |  | 0.162±0.002 |  | 0.181±0.002 |
| Balanced 10 |  |  | 0.057±0.002 |  | 0.030±0.000 |  | 0.167±0.002 |  | 0.180±0.002 |
|  |  |  |  |  |  |  |  |  |  |
| Average |  |  | 0.058±0.002 |  | 0.030±0.000 |  | 0.165±0.002 |  | 0.181±0.002 |
|  |  |  |  |  |  |  |  |  |  |
| Real data |  |  | 0.058±0.002 |  | 0.030±0.000 |  | 0.165±0.002 |  | 0.180±0.002 |
