# Supplementary material for: Clinical Determinants of HIV-1B Between-Host Evolution and their Association with Drug Resistance in Pediatric Patients
Source: PLoS One. 2016 Dec 1;11(12):e0167383. doi: 10.1371/journal.pone.0167383 (PMC5132210; doi:10.1371/journal.pone.0167383)
Supplement: S3 Table — (DOCX) [file pone.0167383.s005.docx]

**S3 Table.** Frequency (%) of sequences with at least one drug resistance mutation in the pediatric HIV-1B population according to patient age.

|  |  | **Age category (years)** | | | |
| --- | --- | --- | --- | --- | --- |
| **DRMs** | **ART**  **experience** | **0-2**  **(n=30)** | **2-8**  **(n=41)** | **8-13**  **(n=50)** | **13-21**  **(n=42)** |
| PI | Naïve | 0 | 0 | 0 | 33 |
|  | Treated | 31 | 36 | 32 | 41 |
|  | All | 13 | 28 | 32 | 67 |
|  |  |  |  |  |  |
| NRTI | Naïve | 13 | 22 | 33 | 33 |
|  | Treated | 31 | 67 | 71 | 71 |
|  | All | 20 | 28 | 32 | 38 |
|  |  |  |  |  |  |
| NNRTI | Naïve | 13 | 22 | 33 | 33 |
|  | Treated | 39 | 43 | 49 | 55 |
|  | All | 23 | 47 | 48 | 40 |

Categories of drug resistance mutations (DRMs) = PI: protease inhibitors; NRTI: nucleoside reverse transcriptase inhibitors; NNRTI: non-nucleoside reverse transcriptase inhibitors.
