# Supplementary material for: Clinical Determinants of HIV-1B Between-Host Evolution and their Association with Drug Resistance in Pediatric Patients
Source: PLoS One. 2016 Dec 1;11(12):e0167383. doi: 10.1371/journal.pone.0167383 (PMC5132210; doi:10.1371/journal.pone.0167383)
Supplement: S4 Table — (DOCX) [file pone.0167383.s006.docx]

**S4 Table.** Estimates of evolutionary parameters for the ten “balanced” replicates (n=30) and the complete (n=133) partial *pol* datasets from the HIV-1B population infecting treated children.

|  |  | ***d*** |  | ***d_N_*** |  | ***d_S_*** |  | ***d_N_/d_S_*** |
| --- | --- | --- | --- | --- | --- | --- | --- | --- |
| Balanced 1 |  | 0.068±0.003 |  | 0.046±0.001 |  | 0.152±0.002 |  | 0.303±0.005 |
| Balanced 2 |  | 0.060±0.004 |  | 0.039±0.001 |  | 0.143±0.002 |  | 0.271±0.004 |
| Balanced 3 |  | 0.059±0.003 |  | 0.037±0.001 |  | 0.141±0.002 |  | 0.261±0.005 |
| Balanced 4 |  | 0.063±0.001 |  | 0.041±0.001 |  | 0.148±0.002 |  | 0.275±0.004 |
| Balanced 5 |  | 0.062±0.004 |  | 0.036±0.001 |  | 0.159±0.002 |  | 0.229±0.003 |
| Balanced 6 |  | 0.055±0.004 |  | 0.034±0.001 |  | 0.136±0.002 |  | 0.248±0.005 |
| Balanced 7 |  | 0.066±0.003 |  | 0.043±0.001 |  | 0.155±0.002 |  | 0.277±0.005 |
| Balanced 8 |  | 0.061±0.004 |  | 0.039±0.001 |  | 0.146±0.002 |  | 0.267±0.004 |
| Balanced 9 |  | 0.060±0.003 |  | 0.038±0.001 |  | 0.141±0.002 |  | 0.272±0.004 |
| Balanced 10 |  | 0.058±0.005 |  | 0.036±0.001 |  | 0.142±0.002 |  | 0.250±0.004 |
|  |  |  |  |  |  |  |  |  |
| Average |  | 0.061±0.001 |  | 0.039±0.001 |  | 0.146±0.002 |  | 0.265±0.006 |
|  |  |  |  |  |  |  |  |  |
| Real data |  | 0.061±0.001 |  | 0.039±0.001 |  | 0.147±0.002 |  | 0.262±0.006 |
